# Incidence and risk factors of intraoperative hyperglycemia in non-diabetic patients: a prospective observational study

**DOI:** 10.1186/s12871-022-01829-9

**Published:** 2022-09-10

**Authors:** Varunya Sermkasemsin, Mali Rungreungvanich, Worapot Apinyachon, Inthuon Sangasilpa, Wanlee Srichot, Chawika Pisitsak

**Affiliations:** Department of Anesthesiology, Faculty of Medicine, Ramathibodi Hospital, Mahidol University, Rama VI Road, Bangkok, 10400 Thailand

**Keywords:** Hyperglycemia, Anesthesia, Outcome, Risk

## Abstract

**Background:**

Intraoperative hyperglycemia has been associated with multiple postoperative complications such as surgical site infection, myocardial infarction, stroke, and death. These complications are not confined to only diabetic patients. However, the incidence of intraoperative hyperglycemia in non-diabetic patients has not been fully elucidated. Additionally, these patients’ risk factors were not well established in previous studies.

**Methods:**

Four hundred forty non-diabetic patients who underwent intermediate- to high-risk surgery were included in the study. We prospectively measured the capillary blood glucose level in all patients during surgery. The incidence of intraoperative hyperglycemia was defined as at least one episode of blood glucose level of more than 180 mg/dL. Risk factors for hyperglycemia were assessed using multivariable logistic regression analysis.

**Results:**

Sixty-five (14.7%) patients developed hyperglycemia during surgery. The independent risk factors for intraoperative hyperglycemia were an American Society of Anesthesiologists status ≥ 3 (odds ratio [OR] 6.09, 95% confidence interval [CI]: 2.67–13.89, *p* < 0.001), preoperative impaired fasting blood sugar (OR 2.28, 95%CI:1.13–4.61, *p* = 0.021), duration of anesthesia ≥ 3 h (OR 4.06, 95%CI: 1.23–13.45, *p* = 0.021), intraoperative hypotension (OR 5.37, 95%CI: 2.35–12.29, *p* < 0.001), intraoperative blood transfusion (OR 4.35, 95%CI: 2.15–8.79, *p* < 0.001), and steroid use (OR 2.39, 95%CI: 1.20–4.76, *p* = 0.013). Surgical site infection was higher in patients with intraoperative hyperglycemia compared with patients without intraoperative hyperglycemia (4 [6.1%] vs. 6 [1.6%], respectively, *p* = 0.035).

**Conclusion:**

The incidence of intraoperative hyperglycemia was significant in non-diabetic patients during intermediate- to high-risk surgery. Risk factors should be identified to prevent intraoperative hyperglycemia.

**Trial registration:**

The study was prospectively registered at https://www.thaiclinicaltrials.org (TCTR20191114001).

## Background

Intraoperative hyperglycemia is associated with multiple postoperative complications such as myocardial infarction, stroke, and postoperative infection [1–6]. However, the impact of intraoperative hyperglycemia has been mainly focused upon in diabetic patients. Intraoperative hyperglycemia and adverse events related to hyperglycemia have been shown in both patients with and without diabetes. There is a dose–response relationship between blood glucose levels and complications [3]. Patients with hyperglycemia who received insulin had an equal risk of adverse events compared with those with normal blood glucose levels [7]. Therefore, treating hyperglycemia with insulin is the key to reducing intraoperative adverse events. However, non-diabetic patients were less likely to receive treatment with insulin compared with diabetic patients for the same blood sugar level. This is because the incidence of intraoperative hyperglycemia is underestimated in non-diabetic patients. The incidence of intraoperative hyperglycemia in non-diabetic patients was reported to be as low as about 6% in some studies [3, 8], but many other studies reported a higher incidence [9–11]. This study aimed to demonstrate the incidence of intraoperative hyperglycemia in non-diabetic patients who underwent intermediate- to high-risk surgery. Risk factors were also identified to prevent intraoperative hyperglycemia.

## Methods

We conducted this prospective observational study between November 2019 and September 2020. After providing written informed consent, adult patients over 18 years of age who were receiving general anesthesia for an intermediate- to high-risk surgery were enrolled. Definition of intermediate-to high-risk surgery was followed a guideline from European Society of Cardiology and European Society of Anaesthesiology [12]. Exclusion criteria were diabetic patients and patient with no history of blood sugar or hemoglobin A1C (HbA1C) within 1 year before surgery. The study was approved by the Institutional Review Board at Mahidol University, Bangkok, Thailand (COA.MURA2019/823). The study was performed based on the Declaration of Helsinki. The trial was registered at https://www.thaiclinicaltrials.org. The first registration date was November 14^th^, 2019 and the register number was TCTR20191114001. The manuscript was written following the STROBE statement for observational studies.

All patients underwent standard general anesthesia procedures on the day of surgery. All cases were monitored with automatic non-invasive blood pressure monitoring, electrocardiography and pulse oximetry. The decision to perform invasive blood pressure and central venous pressure monitoring were depended on attending anesthesiologist. The choices of anesthetic agents were not controlled. Preoperative HbA1C values or fasting blood sugar (FBS) levels were reviewed to rule out pre-existing undiagnosed diabetes mellitus (FBS ≥ 126 mg/dL or HbA1C ≥ 6.5%). We collected capillary blood glucose level information after induction of anesthesia and at the end of surgery. The capillary blood glucose level could have been obtained more frequently in accordance with the attending anesthesiologist preference. Capillary blood was drawn from the tip of patient’s finger. The glucose concentration was determined in fresh capillary blood by reflectance photometry using an Accu-Check Inform II system (Roche, United States). The highest blood glucose level was recorded for the analysis. Hyperglycemia was defined as a blood glucose level of more than 180 mg/dL [13].

Data included age, sex, body mass index, co-morbidities, American Society of Anesthesiologists (ASA) physical status, preoperative FBS and HbA1C levels, intraoperative fluid, blood loss, vital signs, body temperature, steroid use, and blood transfusion were recorded. Hypothermia was defined as a body temperature of less than 36 °C at least one time during surgery. Intraoperative hypotension was defined as having a mean arterial pressure of less than 65 mmHg at least one time during surgery. Impaired FBS was defined as glucose level of 100–125 mg/dL. The patients were followed-up for 30 days after surgery to identify the incidence of surgical site infection (SSI). The definition of surgical site infection was defined according to the United States Centers for Disease Control and Prevention (CDC) [14]. CDC describes 3 types of SSI which were superficial incisional SSI, deep incisional SSI and organ or space SSI.

### Outcomes

The primary outcome was the incidence of intraoperative hyperglycemia in non-diabetic patients. The secondary outcomes were independent risk factors for intraoperative hyperglycemia in non-diabetic patients and the incidence of surgical site infection in patients with intraoperative hyperglycemia.

### Statistical analysis

The incidence of intraoperative hyperglycemia is shown using the frequency and percentage. Patients were divided into a hyperglycemic group and a non-hyperglycemic group to identify the risk factors for intraoperative hyperglycemia. Normality of all continuous data was assessed using the Kolmogorov–Smirnov test. Normally distributed data were presented using the mean ± standard deviation, whereas the median with interquartile range was used for non-normally distributed data. A Kruskal–Wallis or Mann–Whitney U test was used for continuous data, and a chi-square test or Fisher’s exact test was used for categorical variables. Multivariable logistic regression analysis was used to identify the independent risk factors for intraoperative hyperglycemia. Variables with a *p*-value of less than 0.1 from a univariate analysis or variables that had a clinically relevant risks of hyperglycemia were included in the models. Akaike information criterion (AIC) stepwise analysis was used to select the best model for multivariable logistic regression analysis. A *p*-value of less than 0.05 was considered to be statistically significant. Statistical analysis was performed using SPSS version 22.0 (IBM Corp., Armonk, NY, USA).

### Sample size calculation

The incidence of intraoperative hyperglycemia from the previous study was 20% [10]. According to the formula for testing for one population proportion, the reference value (p0), proportion (p), alpha, Z (0.97), beta, Z (0.80) is 0.20, 0.15, 0.05, 1.95, 0.20, 0.84, respectively. Finally, the calculated sample size (n) was 401. The total sample size was increased to 440 cases to prevent data loss.

## Results

**Fig. 1:**
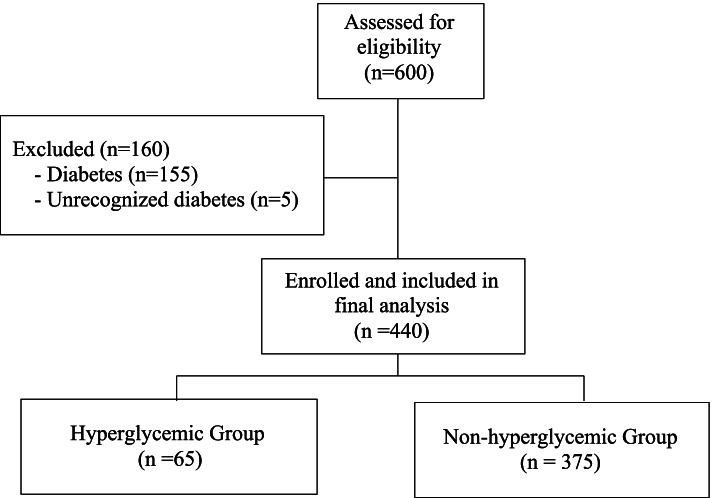
The study flow diagram

The total of 600 patients were assessed for eligibility for the study. One hundred and fifty five patients were excluded because of history of diabetes. Five patients were excluded because of the preoperative FBS higher than 126 mg/dL. Finally, 440 patients were enrolled into the study (Fig. 1). There was no missing data in this study. Patient characteristics in this study are shown in Table 1. The average age was 55.88 ± 15.38 years. There were 45.5% of patients who had an ASA physical status ≥ 3. The patients in this study had an average pre-operative blood sugar level of 83.89 ± 35.6 mg/dL. Combined epidural-general anesthesia technique were used in 36 cases.

**Table 1:** Patient baseline characteristics

	**All patients (*N* = 440)**
Age (years), mean ± SD	55.88 ± 15.38
Female sex, n(%)	279 (63.40)
BMI (kg/m^2^), mean ± SD	24.13 ± 4.16
ASA status≥ 3, n(%)	200 (45.45)
Preoperative FBS (mg/dL), mean ± SD	83.89 ± 35.60
Co-morbidities, n(%)	
Hypertension	169 (38.40)
Coronary artery disease	26 (5.90)
Chronic kidney disease	27 (6.13)
Cerebrovascular accident	18 (4.09)
Cirrhosis	2 (0.45)
Malignancy	105 (23.86)
Chronic obstructive pulmonary disease	9 (2.04)
Emergency surgery	27 (6.13)

*ASA* American Society of Anesthesiologists, *FBS* Fasting blood sugar, *BMI* Body mass index, *SD* Standard deviation

The incidence of hyperglycemia in this study was 14.7%. Patients were divided into a hyperglycemic group (*n* = 65 cases) and a non-hyperglycemic group (*n* = 375 cases) (Table 2). The median (IQR) of blood glucose level for hyperglycemic group and non-hyperglycemic group were 200 (189–225) mg/dL and 133 (114–150) mg/dL, respectively. Most of the patients in this study underwent major gastrointestinal surgery (*n* = 121, 27.50%), neurosurgery (*n* = 91, 20.68%), or gynecological surgery (*n* = 77, 17.50%). Only cardiovascular thoracic (CVT) surgery was associated with increased intraoperative hyperglycemia [hyperglycemic group *n* = 20 (30.76%) vs. non-hyperglycemic group *n* = 10 (2.66%), OR (95% CI) 18.07 (7.76–42.09), *p* < 0.001] (Table 3).

**Table 2:** Univariate analysis for intraoperative hyperglycemia

	Hyperglycemic group (*N* = 65)	Non-hyperglycemic group (*N *= 375)	OR (95% CI)	*p*-value
Patient characteristics				
Age ≥ 65 years, n(%)	28 (43.07)	111 (29.60)	1.80 (1.05–3.08)	0.031
Female sex, n(%)	34 (52.30)	245 (65.33)	0.58 (0.34–0.99)	0.044
BMI (kg/m^2^), median (IQR)	22.77 (21.01–25.28)	24.14 (21.48–26.37)	0.93 (0.87–1.00)	0.061
ASA status ≥ 3, n(%)	55 (84.61)	145 (38.67)	8.72 (4.31–17.65)	< 0.001
Preoperative impaired FBS, n(%)	27 (41.53)	118 (31.46)	1.54 (0.90–2.65)	0.111
Co-morbidities, n(%)				
Hypertension	37 (56.92)	132 (35.20)	2.43 (1.42–4.15)	0.001
Coronary artery disease	15 (23.07)	11 (2.93)	9.92 (4.31–22.81)	< 0.001
Chronic kidney disease	7 (10.76)	20 (5.33)	2.14 (0.86–5.29)	0.097
Cerebrovascular accident	7 (10.76)	11 (2.93)	3.99 (1.48–10.71)	0.009
Dyslipidemia	20 (30.76)	91 (24.26)	1.38 (0.77–2.47)	0.265
Cirrhosis	2 (3.1)	0 (0)		0.022
Malignancy	18 (27.7)	87 (23.20)	1.26 (0.70–2.29)	0.433
Chronic obstructive pulmonary disease	2 (3.07)	7 (1.86)	1.66 (0.33–8.21)	0.627
Emergency surgery, n(%)	2 (3.07)	25 (6.66)	0.44 (0.10–1.92)	0.401
Preoperative dextrose solution, n(%)	38 (58.46)	292 (77.86)	0.40 (0.23–0.70)	0.001
Intraoperative dextrose solution, (mL/hour) (mean ± SD)	15.23 ± 44.74	7.85 ± 25.63	1.00 (0.99–1.01)	0.080
Intraoperative isotonic crystalloid solution, (mL/hour) (mean ± SD)	445.75 ± 294.95	468.19 ± 246.20	1.00 (0.99–1.00)	0.514
Total intraoperative crystalloid solution, (L) (mean ± SD)	3.00 ± 2171.60	2.04 ± 1552.99	1.33 (1.15–1.53)	< 0.001
Intraoperative events				
Crystalloid ≥ 2000 mL, n(%)	41 (63.07)	169 (45.06)	2.04 (1.19–3.48)	0.007
Blood transfusion, n(%)	44 (67.69)	62 (16.53)	10.57 (5.88–19.02)	< 0.001
Duration of anesthesia ≥ 3 h, n(%)	61 (93.84)	241 (64.26)	8.47 (3.01–23.83)	< 0.001
Intraoperative hypotension, n (%)	54 (83.07)	119 (31.73)	10.56 (5.33–20.92)	< 0.001
Hypothermia, n(%)	55 (84.61)	248 (66.13)	2.85 (1.40–5.77)	0.003
Steroid use, n(%)	32 (49.23)	125 (33.33)	1.93 (1.14–3.30)	0.014

Statistical significance was set at *p* < 0.05

*ASA* American Society of Anesthesiologists, *FBS* Fasting blood sugar, *ENT* Ear Nose Throat, *MAP* Mean arterial pressure, *BMI* Body mass index, *SD* Standard deviation

**Table 3:** Types of surgery

Surgical procedure	Hyperglycemic group (*N* = 65)	Non-hyperglycemic group (*N* = 375)	*P*-value
Abdominal surgery, n(%)	22 (33.84)	99 (26.40)	0.215
Cardiothoracic surgery, n(%)	20 (30.76)	10 (2.66)	< 0.001
Vascular surgery, n(%)	4 (6.15)	17 (4.53)	0.532
Reconstructive surgery, n(%)	4 (6.15)	11 (2.93)	0.254
Urological surgery, n(%)	5 (7.69)	33 (8.80)	0.769
Neurosurgery, n(%)	5 (7.69)	86 (22.93)	0.005
Obstetrics & gynecological surgery, n(%)	4 (6.15)	73 (19.46)	0.009
Orthopedic surgery, n(%)	0 (0)	4 (1.06)	1.000
Head and neck surgery, n(%)	1 (1.53)	42 (11.20)	0.015

**Fig. 2:**
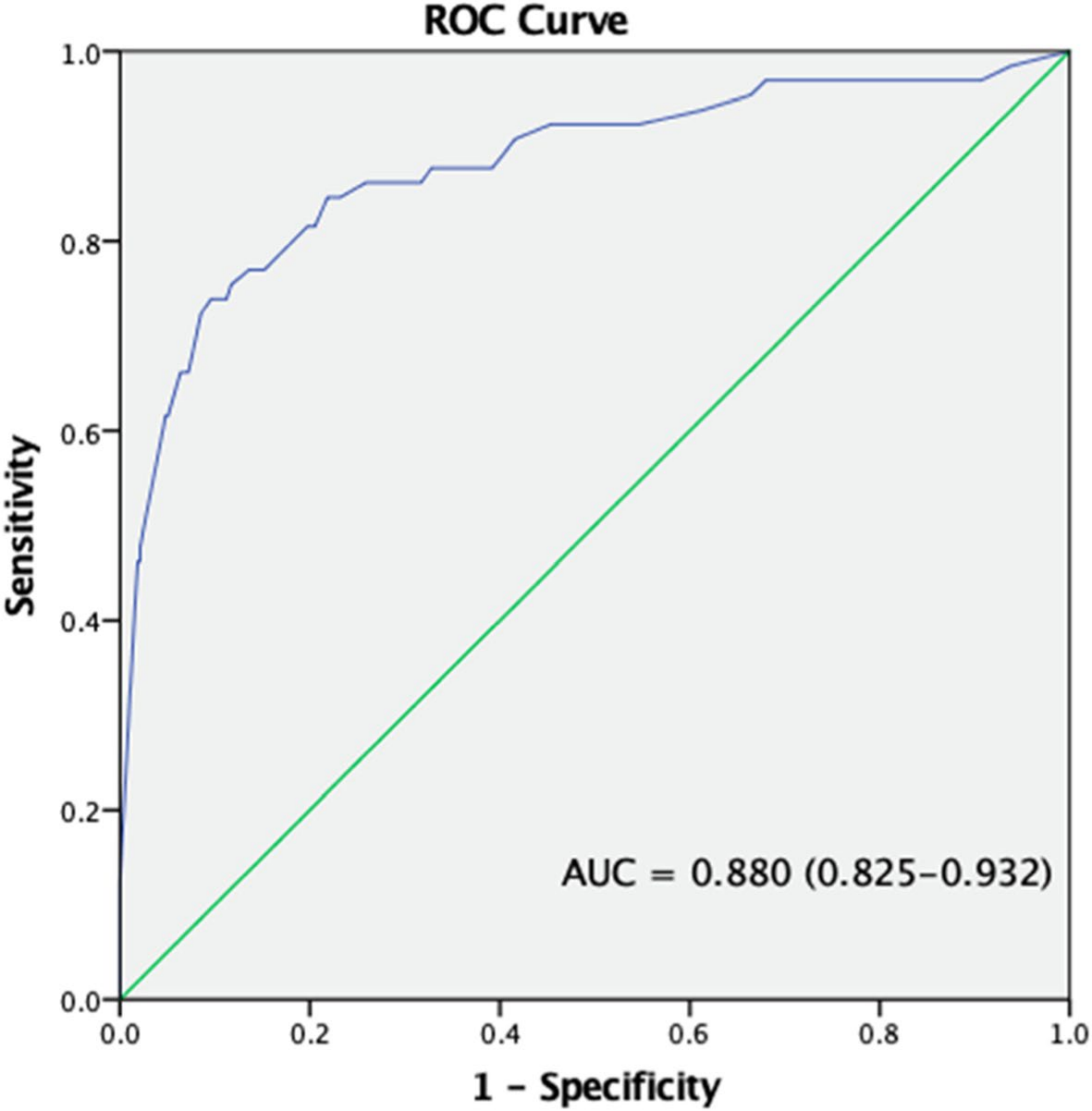
Receiver operating characteristic curve of the final model of multivariable logistic regression analysis

Variables selected for inclusion into the model were age, sex, preoperative impaired FBS, ASA physical status, anesthesia duration ≥ 3 h, intraoperative hypotension, intraoperative blood transfusion, intraoperative steroid use, intraoperative hypothermia, and crystalloid use > 2 L. ASA physical status had a high correlation with co-morbidities and body mass index. Therefore, only ASA physical status was included in the model. Also, types of surgery were associated with other perioperative factors, for instance, history of blood transfusion, duration of anesthesia. Therefore, types of surgery were not included in the model. The best-fit model, carrying 75% of the cumulative model weight, included six parameters with no interaction effect. The sensitivity, specificity, the area under a receiver operating characteristic (ROC) curve of final model with lowest AIC were 60.00%, 95.12%, 0.88, respectively (Fig. 2). The independent risk factors for intraoperative hyperglycemia were ASA status ≥ 3 (odds ratio [OR] 6.09, 95% confidence interval [CI]: 2.67–13.89, *p* < 0.001), preoperative impaired FBS (OR 2.28, 95%CI:1.13–4.61, *p* = 0.021), anesthesia duration ≥ 3 h (OR 4.06, 95%CI: 1.23–13.45, *p* = 0.021), intraoperative hypotension (OR 5.37, 95%CI: 2.35–12.29, *p* < 0.001), intraoperative blood transfusion (OR 4.35, 95%CI: 2.15–8.79, *p* < 0.001), and steroid use (OR 2.39, 95%CI: 1.20–4.76, *p* = 0.013) (Table 4).

**Table 4:** Multivariable logistic regression analysis for intraoperative hyperglycemia

Variables	Adjusted OR (95%CI)	*P*-value
ASA status≥ 3	6.09 (2.67–13.89)	< 0.001
Preoperative impaired FBS	2.28 (1.13–4.61)	0.021
Duration of anesthesia≥ 3 h	4.06 (1.23–13.45)	0.021
Intraoperative hypotension	5.37 (2.35–12.29)	< 0.001
Blood transfusion	4.35 (2.15–8.79)	< 0.001
Steroid use	2.39 (1.20–4.76)	0.013

Statistical significance was set at *P* < 0.05

*ASA* American Society of Anesthesiologists, *FBS* Fasting blood sugar, *OR* Odds ratio, *95% CI* 95% confidence interval

There were three types of steroid use in this study. The most commonly used steroid was dexamethasone (136 cases, 30.90%), with an average dose of 8 mg per patient. Hydrocortisone was used in 14 patients (3.18%), with a median dose of 50 mg. Methylprednisolone was used in seven patients (1.59%), with a median dose of 2300 mg.

The surgical site infection at 30 days after surgery was higher in patients with intraoperative hyperglycemia than in patients without intraoperative hyperglycemia [4 (6.1%) vs. 6 (1.6%), OR 4.03 (95% CI 1.10–14.70), *p* = 0.035].

## Discussion

The incidence of hyperglycemia in non-diabetic patients has likely been under-estimated because it has a lower incidence compared with that of diabetic patients. The incidence of intraoperative hyperglycemia in non-diabetic patients in this study was 14.7%. Similarly, Potisuk et al. reported an incidence of 15% for intraoperative hyperglycemia during abdominal surgery in non-diabetic patients [11]. The incidence in this study was much lower than the incidence of 40% in diabetic patients that was reported by Kotagal et al. [3]. Because they likely have a lower incidence of hyperglycemia, intraoperative blood glucose in non-diabetic patients is monitored less frequently than that in diabetic patients. However, some studies reported a higher incidence of intraoperative hyperglycemia in non-diabetic patients. Han et al. reported an incidence of 35.5% for intraoperative hyperglycemia during liver resection [9], whereas Bhattacharjee et al. reported an incidence of 20% in neurosurgery for traumatic brain injury [10], which shows the variability in the results. Although we found a lower incidence of intraoperative hyperglycemia than in these above-mentioned studies, the increased incidence of surgical site infection was observed. Similarly, previous studies showed a high incidence of surgical site infection [4] and anastomotic leakage in colorectal surgery after intraoperative hyperglycemia [5]. Furthermore, Kotagal et al. reported that hyperglycemic non-diabetic patients have higher odds of adverse events occurring than those with hyperglycemic diabetes [3]. This phenomenon was called the diabetes paradox.

Previous studies also demonstrated the relationship between a tighter control of intraoperative blood glucose and lower intraoperative adverse events. Mansur et al. found that blood glucose levels lower than 150 mg/dL during cardiac surgery with cardiopulmonary bypass were associated with decreased 5-year mortality [15]. De Vries et al. also proposed that keeping the blood glucose lower than 150 mg/dL during the intraoperative period could reduce surgical site infection. Although the risk of hypoglycemia was higher, they reported no serious adverse events related to hypoglycemia [16]. Similarly, Kotagal et al. demonstrated a dose–response relationship between the blood glucose level and composite adverse events (OR, 1.3 for blood glucose 125–180 mg/dL, 95% CI, 1.1–1.5; OR, 1.6 for blood glucose 180 mg/dL, 95% CI, 1.3–2.1). From the above findings, there should be more intraoperative blood glucose monitoring and treatment for non-diabetic patients.

We found that impaired FBS was associated with intraoperative hyperglycemia. Biker et al. found that patients with preoperative impaired FBS who underwent major surgery had more intraoperative cardiovascular events compared with those with normal FBS, such as in acute coronary syndrome or acute heart failure [17]. Similarly, Davies et al. found that patients with impaired FBS who underwent carotid artery stenting had more major adverse events (i.e., death, myocardial infarction, or stroke) compared with those without FBS impairment [18]. These results emphasized the clinical significance of preoperative impaired FBS. Our findings suggest that the mechanism for the increased incidence of cardiovascular events might be the increased intraoperative hyperglycemia in this group of patients. Future research should be performed to assess the need to postpone elective surgery until preoperative blood glucose is controlled.

We found that intraoperative steroid use in non-diabetic patients increased the incidence of hyperglycemia, which is consistent with results from other studies [19, 20]. However, this finding was different from the previous study by Corcoran et al., which described that 4 or 8 mg of dexamethasone did not cause intraoperative hyperglycemia [21]. Although there have been conflicting results about the relationship between intraoperative steroid use and hyperglycemia, there is no evidence of complications after steroid use. Polderman et al. demonstrated that dexamethasone did not cause postoperative wound infection [22]. Toner et al. found that dexamethasone was associated with hyperglycemia but not increased infection incidence or length of hospital stay [23]. Thus, there is currently no recommendation to withhold steroid use to control intraoperative blood glucose. However, capillary blood glucose should be monitored after steroid administration.

All other risk factors including high ASA physical status, long surgical duration, blood transfusion, and intraoperative hypotension were related to stress hyperglycemia, which is defined by patients who developed hyperglycemia while critically ill or during surgery without a previous diabetes mellitus diagnosis. The mechanism of stress hyperglycemia was explained by excessive gluconeogenesis, glycogenolysis, and insulin resistance. These neuroendocrine responses were triggered by stress, which stimulated the hypothalamic–pituitary–adrenal axis, sympathoadrenal system, and proinflammatory cytokines (tumor necrosis factor-α, interleukin (IL)-1 and IL-6) [24, 25]. Therefore, all of the above risk factors indicate that the anesthesiologist should monitor intraoperative blood glucose in nondiabetic patients.

According to the result of this study, blood glucose should be monitored in non-diabetic patient undergoing intermediate-to high-risk surgery especially patients with risk factors. Some factors are modifiable, therefore, intraoperative hyperglycemia was preventable in many cases.

There were some limitations in this study. First, the blood glucose was not monitored at regular intervals. Therefore, the incidence of hyperglycemia might be underestimated in intermediate-risk surgery compared with high-risk surgery because anesthesiologists tend to collect blood glucose more frequently during high-risk surgeries such as cardiothoracic surgery or neurosurgery compared with lower-risk surgeries. Second, blood glucose was not monitored in non-diabetic patients before surgery in our current practice, and we could not assess the blood glucose level before anesthesia induction because there was a concern about ethical issues. Thus, we could not demonstrate the impact of preoperative blood glucose on intraoperative hyperglycemia. FBS was assessed only at the preoperative visit.

## Conclusion

There was a significant incidence of intraoperative hyperglycemia in non-diabetic patients during intermediate- to high-risk surgery. Blood glucose levels in patients with risk factors for intraoperative hyperglycemia should be monitored closely.

## Data Availability

The data of this study could be requested from the corresponding authors.
